# Crotoxin from *Crotalus durissus terrificus* Is Able to Down-Modulate the Acute Intestinal Inflammation in Mice

**DOI:** 10.1371/journal.pone.0121427

**Published:** 2015-04-08

**Authors:** Caroline de Souza Almeida, Vinicius Andrade-Oliveira, Niels Olsen Saraiva Câmara, Jacqueline F. Jacysyn, Eliana L. Faquim-Mauro

**Affiliations:** 1 Laboratory of Immunopathology, Butantan Institute, São Paulo, São Paulo, Brazil; 2 Department of Immunology, University of São Paulo, São Paulo, Brazil; 3 LIM62—Faculty of Medicine, University of São Paulo, São Paulo, Brazil; Seoul National University College of Pharmacy, KOREA, REPUBLIC OF

## Abstract

Inflammatory bowel diseases (IBD) is the result of dysregulation of mucosal innate and adaptive immune responses. Factors such as genetic, microbial and environmental are involved in the development of these disorders. Accordingly, animal models that mimic human diseases are tools for the understanding the immunological processes of the IBD as well as to evaluate new therapeutic strategies. Crotoxin (CTX) is the main component of *Crotalus durissus terrificus* snake venom and has an immunomodulatory effect. Thus, we aimed to evaluate the modulatory effect of CTX in a murine model of colitis induced by 2,4,6- trinitrobenzene sulfonic acid (TNBS). The CTX was administered intraperitoneally 18 hours after the TNBS intrarectal instillation in BALB/c mice. The CTX administration resulted in decreased weight loss, disease activity index (DAI), macroscopic tissue damage, histopathological score and myeloperoxidase (MPO) activity analyzed after 4 days of acute TNBS colitis. Furthermore, the levels of TNF-α, IL-1β and IL-6 were lower in colon tissue homogenates of TNBS-mice that received the CTX when compared with untreated TNBS mice. The analysis of distinct cell populations obtained from the intestinal lamina propria showed that CTX reduced the number of group 3 innate lymphoid cells (ILC3) and Th17 population; CTX decreased IL-17 secretion but did not alter the frequency of CD4^+^Tbet^+^ T cells induced by TNBS instillation in mice. In contrast, increased CD4^+^FoxP3^+^ cell population as well as secretion of TGF-β, prostaglandin E_2_ (PGE_2_) and lipoxin A_4_ (LXA_4_) was observed in TNBS-colitis mice treated with CTX compared with untreated TNBS-colitis mice. In conclusion, the CTX is able to modulate the intestinal acute inflammatory response induced by TNBS, resulting in the improvement of clinical status of the mice. This effect of CTX is complex and involves the suppression of the pro-inflammatory environment elicited by intrarectal instillation of TNBS due to the induction of a local anti-inflammatory profile in mice.

## Introduction

Immune tolerance is responsible for controlling inflammation in the gastrointestinal tract, limiting the response against antigens derived from food and commensal bacteria [1, 2, 3]. However, a breakdown in this tolerogenic status due to distinct factors such as genetic or environmental can result in a dysregulated immunological response and consequent inflammatory bowel disease (IBD) [4, 5, 6, 7, 8].

Crohn's disease and ulcerative colitis are two major forms of inflammatory bowel disease (IBD). Macrophages secreting high levels of TNF-α and IL-1 as well as activated neutrophils are involved in the pathogenesis of these diseases [9, 10]. Group 3 of innate lymphoid cells (ILC3) have also been described as a crucial cell population for protective immunity in the intestinal environment [11]. The ILC3 are lineage marker-negative (LIN^-^) cells that do not express a T cell receptor and are characterized by the expression of transcription factor RORγt [12]. The IL-22 is their cytokine marker mediating distinct functions such as epithelial cells activation in the intestinal tissue [13]. Secretion of IL-17A as well as IL-22 by this cell population has been implicated in the intestinal immunity to enteric pathogens [14, 15]. Furthermore, ILC3 secreting IL-17A are involved in the inflammation observed in distinct models of IBD [16, 17].

Adaptive immune response in Crohn's disease is mediated by Th1 cells secreting IL-12 and IFN-γ. Furthermore, Th17 lymphocytes expressing RORγt have also been observed in the lamina propria of patients with this disease [18, 19, 20]. In contrast, the ulcerative colitis is correlated with the Th2 cells secreting high levels of cytokines, such as IL-13 and IL-5 [21].

Accordingly, distinct cell populations secreting cytokines are essential for the activation or maintenance of homeostasis of the immune system in the mucosal environment. Therefore, secreted products in distinct phases of the immune response mediate activation or exert inhibitory effects on different cell populations. In the axis of the regulatory cytokines, IL-10 modulates the functional activity of antigen-presenting cells (APCs) and consequent T cell differentiation [22, 23]. The presence of TGF-β, together with IL-10 has also been associated with tolerance induction and generation of Treg cells [23, 24]. In addition, prostaglandin E_2_ (PGE_2_) and lipoxin A_4_ (LXA_4_), eicosanoids produced from arachidonic acid degradation, have been described as potent modulators of inflammation, APCs activity and cellular immune response [25, 26, 27, 28, 29].

The spontaneous development of colitis in IL-10 deficient mice shows the relevance of this cytokine in controlling the immune response to commensal flora of the gut [30]. TGF-β secreted by regulatory T cells has also been shown to participate in the prevention of colitis by several mechanisms [31, 32, 33]. Therefore, considering the complexity of IBD the murine models have become useful tools to clarify the mechanisms involved in the exacerbated immune response in these diseases as well as to characterize molecules that are able to modulate this mucosal inflammation [34]. In this sense, the most commonly used murine models of intestinal inflammation are those chemically induced by dextran sodium sulfate and 2,4,6-trinitrobenzene sulfonic acid (TNBS) [35, 36].

The venom of *Crotalus durissus terrificus* (*C*.*d*.*terrificus*) snakes is composed by many molecules that mediate important biological activities, where the crotoxin (CTX) is the most abundant toxin. CTX is a heterodimer of approximately 24kDa comprising two polypeptide chains: one acidic, non-toxic and non-enzymatic, called crotapotin (CA), and the another basic, the subunit CB, a phospholipase A_2_ (PLA_2_) that exhibits the toxic activity [37, 38, 39]. The CA subunit behaves as a chaperone, directing CB to its target where the CTX exerts its activity [40, 41].

*C*.*d*. *terrificus* venom has been shown to induce a down-modulation of the immune system resulting in low anti-crotalic antibody production compared with other snake venoms [42, 43, 44]. This suppressive effect of *C*.*d*. *terrificus* venom was also verified in the humoral response induced by unrelated antigens and its effect was mediated by CTX [45]. In addition, it was reported that splenic cell proliferation and cytokine secretion where inhibited in mice injected with whole venom or CTX [45, 46]. CTX was also able to exert a potent inhibitory effect on humoral and cellular responses induced by human serum albumin (HSA) immunization even when injected after the elicitation of innate immunity [47]. *C*.*d*.*terrificus* venom, CTX and CB subunit also have potent modulatory effects on inflammatory cells activity [48, 49, 50].

Considering that the CTX from *C*.*d*.*terrificus* venom exerts a potent immunomodulatory effect, we investigated in this work the ability of this toxin to interfere with the development of the murine model of acute colitis induced by TNBS. Colitis model in mice was determined by disease activity index (DAI), macroscopic tissue damage, histopathological score and myeloperoxidase (MPO) activity. Furthermore, we evaluated the secretion of pro- and anti-inflammatory cytokines in colonic tissue homogenates as well as the ILC3 and T cell populations in the lamina propria from each group of mice. Our data demonstrated that the CTX ameliorates the inflammatory intestinal reaction, improving the clinical score of the disease and inhibiting the secretion of pro-inflammatory cytokines in mice. Furthermore, the CTX was able to down-modulate ILC3 and Th17 immune response and to induce the secretion of anti-inflammatory mediators and CD4^+^Foxp3^+^ cells in mice with TNBS-induced colitis.

## Material and Methods

### Animals

Male BALB/c mice with 7–8 weeks of age were bred in the animal house facilities of the Butantan Institute, São Paulo, Brazil. All experiments using mice were reviewed and approved by the Institutional Animal Care Committee of Butantan Institute (CEUAIB, protocol No. 719/2010) and Ethical Committee for Animal Research of the Institute of Biomedical Sciences/University of São Paulo (CEEA, protocol No. 80). The mice were acclimatized for one week before any experimental procedures under controlled temperature (25°C) with a 12/12 h light-dark cycle and standard feed and water *ad libitum*. All efforts were made to minimize animal suffering. The animals were euthanized using an overdose of anesthesia on the 4th day of TNBS induction.

### Crotoxin isolation

A single-step purification process by anion exchange chromatography was used to isolate CTX from crude venom [44, 51]. Lyophilized crude *C*.*d*.*terrificus* venom was from a pool obtained from several specimens of *C*.*d*.*terrificus* snakes and supplied by the Herpetology Laboratory, of Butantan Institute. Samples of *C*.*d*.*terrificus* (15 mg) were dissolved in 2.0 mL of Tris-HCl buffer (50 mM, pH 7.3) and centrifuged at 10,000 x g for 10 min (Ultra-Centrifuge, Eppendorf). The supernatants were subjected to anion exchange chromatography using a Mono-Q HR 5/5 column in an Akta-FPLC system (GE Healthcare; Uppsala, Sweden). Proteins were eluted from the column by a linear gradient of 0–1 M NaCl in 50 mM Tris-HCl, pH 8.3. Fractions of 1.0 mL/tube were collected and the chromatographic profile was determined by absorbance at 280 nm. Three distinct peaks were obtained during the purification process, and crotoxin was eluted in the 2^nd^ peak [51]. The homogeneity of CTX was confirmed by non-reducing SDS-PAGE (12.5%). The protein content was measured by the Bradford method (1976) [52].

The CTX stock solution was subjected to chromatographic separation using a polymyxin B column (Pierce Biotechnology, Rockford, IL) to reduce endotoxin content. The endotoxin level in the purified CTX solution was less than 1 (EU) per milligram of protein as determined with a *Limulus amebocyte* lysate kit (Sigma Chemical Co., St. Louis, MO).

### Induction of acute intestinal inflammation in mice and CTX administration

Groups of mice were anesthetized with saline solution containing ketamine (100 mg/kg) and xylazine (10 mg/kg) given ip. Intestinal inflammation was induced by the administration of 2.5 mg of TNBS dissolved in 45% ethanol (100 μL/animal). The TNBS solution was administered via a 3.5 F catheter coupled to a syringe of 1-mL through the rectum into the colon until the tip was 4 cm proximal to the anus [53, 54]. The mice were held by the tail in a vertical position for 1 min after the intrarectal administration to ensure retention of the haptenating agent within the entire colon and cecum. The control group received 100 μL of a solution containing only 45% ethanol. Eighteen hours (18h) after TNBS administration, the mice were injected i.p. with 300μL of CTX (0.035 mg/kg), and the control group received only 300μL of saline solution.

### Clinical evaluation of mice and sample collection

The clinical evaluation of the inflammatory reaction in mice was performed daily according to the CDAI (Crohn's disease activity index) used for patients with Crohn's disease. The following parameters were used for calculation: a) Weight loss (no loss = 0 point (pt); from 1 to 5% = 1 pt.; 5 to 10% = 2 pts, 10 to 15% = 3 pts; above 15% = 4 pts.); b) Consistency of stools / diarrhea (normal = 0 pts; loose stools = 2 pts; mucus excreted perianally, rectal prolapse and diarrhea = 4 pts). The sum of the parameters weight loss and stool consistency parameters resulted in the CDAI score ranging from 0 (unaffected) to 8 points (severe disease), adapted by Alex et al. (2009) [55].

At the end of 4 days of induction with TNBS, the animals were euthanized, dissected and the entire colon was quickly removed and gently cleared of feces. Small segments of the colon were frozen immediately in liquid nitrogen for the analysis of the cytokine secretions and MPO activity. The colon segments were used for obtaining the lamina propria and preparing the cell suspensions.

### Histology

The perirectal segment (0.5 cm) was collected for histopathological analysis. The tissues were fixed in 10% neutral formalin, dehydrated, embedded in paraffin and sectioned (4-μm thickness). The sections were stained with hematoxylin and eosin (Instant-Prov kit, Newprov, Pinhais, Brazil) and evaluated by light microscopy for the study of tissue inflammation according to a histological score. The following parameters were used for calculation: a) epithelial damage (no damage = 0 pt.; minimal loss of goblet cells = 1pt; extensive loss of goblet cells = 2 pts; minimal loss of crypts and extensive loss of goblet cells = 3 pts; extensive loss of crypts = 4 pts); b) inflammatory infiltrate (none = 0; infiltrate around crypts = 1 pt; infiltrate in mucosa = 2 pts; infiltrate extensive mucosal edema = 3 pts; infiltrate in submucosa = 4 pts). The histological activity index was calculated by summing the score for tissue damage and inflammatory cell infiltration points ranging from 0 (unaffected) to 8 pts (severe disease), adapted by Alex et al. (2009) [55].

### Determination of the myeloperoxidase (MPO) enzymatic activity

MPO was determined using the method described by Bradley et al. (1982) [56] with modifications. The colonic intestinal portions were homogenized in 50 mM potassium phosphate buffer (pH 6.0) containing 0.5% hexadecyltrimethylammonium bromide (HTAB) (Sigma-Aldrich). The samples were quickly frozen in liquid nitrogen and thawed in warm water at 37°C for 2 min. After repeating this procedure, the samples were centrifuged at 11,000 x g for 10 min at 4°C. The supernatants were collected and 50 μL diluted in potassium phosphate buffer (pH 6.0) containing 0.167 mg O-dianisidine dihydrochloride (Sigma-Aldrich) and 20 mM 30% hydrogen peroxide for determination of enzymatic activity. The tissue protein concentration was also measured for adjustment of MPO values using the "BCA Protein Assay Reagent Kit A and B" (Thermo Scientific). The absorbance read at 460 nm for the MPO reaction mixture and 562 nm for determination of tissue protein. The results were expressed as MPO units/mg of tissue protein.

### Cytokine determination in colonic tissue by ELISA

For cytokine analysis, the colonic tissue was homogenized in a solution containing 10 mM Tris-HCl, 1mM EDTA, 1% Triton X-100 and 2 mM PMSF (phenylmethylsulfonyl fluoride—Sigma). In each sample was added 1 μL/mL of protease inhibitor cocktail (Sigma) followed centrifugation at 11,000 x g for 10 min at 4°C. The supernatants were collected for cytokines assays, adapted by Zang et al. (2006) [54]. Levels of TNF-α, IFN-γ, IL-1β, IL-6, IL-17, IL-10, and TGF-β in the homogenates were determined by sandwich enzyme-linked immunosorbent assay (ELISA) using kits supplied by R&D Systems (Minneapolis, MN, USA). ELISA was performed according to the manufacturer’s instructions. Briefly, we used polyclonal goat anti-mouse cytokine antibodies for capture and biotinylated polyclonal rabbit anti-mouse cytokine antibodies for cytokine detections. Streptavidin-HRP conjugate and tetramethylbenzidine sulfonate plus H_2_O_2_ were used for color development. Sulfuric acid (0.2 M) was added to stop reaction and the plates were read at 450 nm in an automated ELISA reader (Spectramax- Molecular Devices LLC, CA, US). Samples were quantified in serial twofold dilutions by comparison with standard curves of purified recombinant cytokines and the values expressed as the mean of individual samples in pg/mL ± standard error of mean (SEM).

### Measurement of eicosanoids in homogenates of colonic tissue of mice

The secretion of PGE_2_ and LXA_4_ was assayed in the colonic intestinal tissue from different experimental mice groups using a sandwich ELISA kit from R&D Systems and Neogen (Lansing, Michigan, USA), respectively. For this, the portions of the colonic intestinal tissue were collected, macerated and homogenized in diluent reagent supplied by each kit. The samples were then centrifuged at 11,000 x g for 10 min at 4°C and the supernatants were acidified with 1 N HCl pH 3.4–3.6. After this, the samples were subjected to octadecylsilyl silica column chromatography (C_18_Sep-Pak column, Waters Corporation, USA), where the column was pre-washed with 10 mL of absolute ethanol and 10 mL of water. The column was activated with 10 mL of water and 10 mL of ethanol (35%) and the eicosanoid contents were eluted with 2 mL absolute ethanol. The samples were dried under a stream of nitrogen and then used to determine the PGE_2_ and LXA_4_ levels by ELISA. The assays were performed according to the manufacturer’s suggestions. Samples were quantified in serial twofold dilutions by comparison with standard curves of purified recombinant cytokines and the values expressed as the mean of individual samples in ng/mL ± SEM.

### Preparation and isolation of lymphocytes from intestinal lamina propria (LPL)

LPLs were obtained and separated as describe for Davis and Parrot (1981) [57]. Samples of colonic tissue were collected and washed in calcium- and magnesium-free Hank’s balanced salt solution (HBSS, Gibco) free of calcium and magnesium. Afterwards, the samples were then cut into pieces of approximately 5mm and incubated in HBSS with 1 mM EDTA (Sigma) and 1 mM DTT (Sigma) at 37°C under constant stirring for 30 min. The tissue digestions was performed by a second incubation in RPMI1640 medium (Invitrogen) containing 400 U/mL collagenase IV (Gibco) and 0.01 mg/mL DNase I (Sigma Chemical Co.) at 37°C for 1 h with constant stirring. For the lymphocytes isolation, the cell suspensions was centrifuged (500 x g for 20 min at 25°C) on a Percoll gradient (Sigma) and collected in the interface layer to 30–70%. The determination of cell count and viability was performed in a Neubauer chamber using 0.2% Trypan Blue and the total of 10^6^ cells were used for staining with fluorescent antibodies for flow cytometric analysis.

### Analysis of cell populations by flow cytometry

The cell suspensions obtained from lamina propria were incubated with anti-FcγRII/III (1μg/10^6^ cells) for 15 min at 4°C followed by addition of 0.5% bovine serum albumin (BSA), 2 mM EDTA in PBS and centrifuged (500 x g for 5 min at 4°C).

To analyze the IL-17-secreting ILC3, the cell suspensions from lamina propria were stimulated for 4h with 50 ng/mL PMA (Sigma-Aldrich), 1mg/mL ionomycin (Sigma-Aldrich) and monensin (GolgiStop; BD Biosciences) at 37°C. Afterwards, the cells were washed with PBS and incubated with a cocktail of APC-conjugated anti-CD4, anti-CD8, anti-CD3, anti-CD19, anti-NK1.1 and anti-B220 antibodies (BD Pharmingen) to exclude the LIN^+^ cells and with FITC-conjugated anti-CD90 (Thy1.2) and PE-conjugated anti-IL-17 antibodies (BD Pharmingen). Cytofix/Cytoperm (BD) was used to wash and permeabilize the cells for the intracellular staining after surface labeling.

For lymphocyte subsets staining, the cell suspensions from lamina propria were incubated with Pacific-Blue-conjugated anti-CD4, FITC-conjugated anti-TCR-β, PE-conjugated anti-RORγt, PercPCy5-conjugated anti-Tbet or PE-conjugated anti-FoxP3 antibodies (BD Pharmingen). Intranuclear staining for transcription factors RORγt, Tbet and Foxp3 was performed according to the manufacture’s protocol (eBioscience). Staining of Th17 cells was performed with PE-Cy5-conjugated anti-CD4, APC-conjugated anti-TCR-β and PE-conjugated anti-IL-17 antibodies (BD Pharmingen). After incubation, wash buffer was added to each sample, followed by centrifugation at 500 x g for 5 min at 4°C. Afterwards, the samples were fixed with 150 μL of 1% paraformaldehyde in PBS.

All samples were performed in duplicate and analyzed in the flow cytometer (FACSCanto II, Becton Dickinson). The data were analyzed with FlowJo software 7.5.

### Statistical analysis

The results were analyzed using GraphPad Prism 5.00 software, by one-way ANOVA followed by the Tukey test, comparing all experimental groups. Results were expressed as the means ± SEM (n = 4–6 animals/group). *p*<0.05 was considered statistically significant.

## Results

### CTX administration improved clinical symptoms and histopathological changes of acute inflammatory reaction in mice

**Fig 1:**
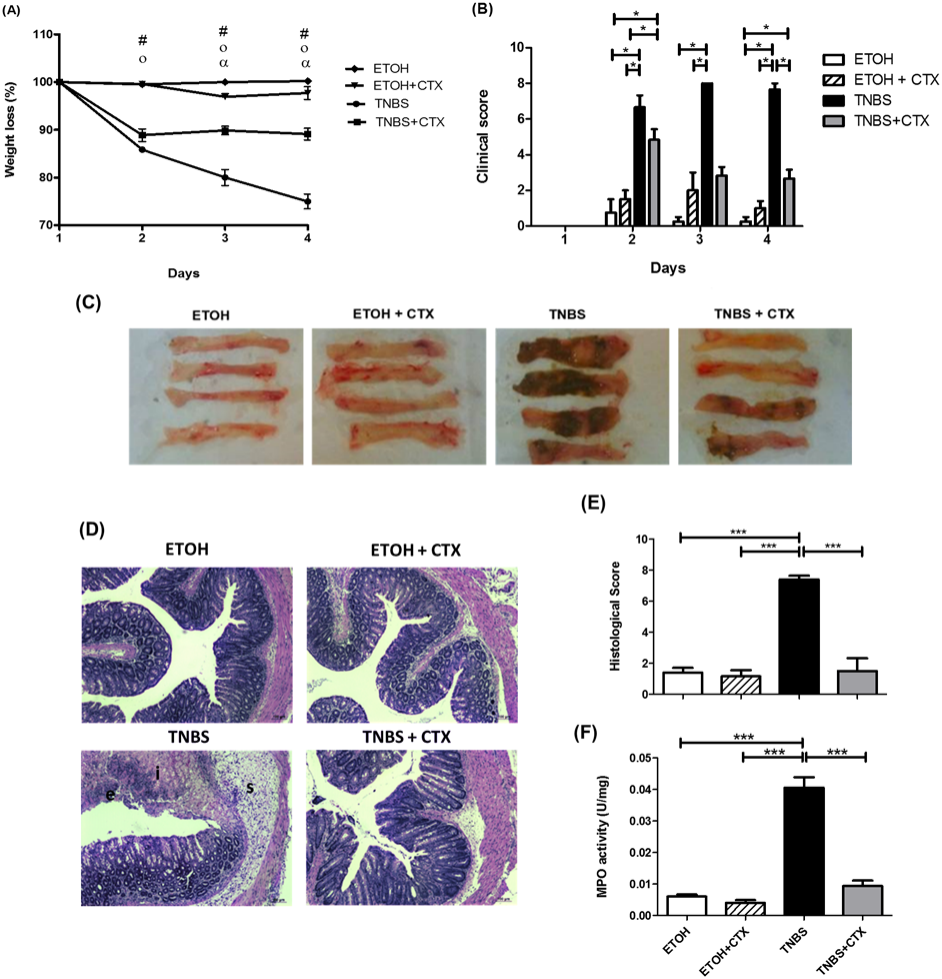
Effect of CTX treatment on the colitis induced by TNBS instillation in BALB/c mice. **(A)** Body weight changes (%) of BALB/c mice during four days after the intrarectal instillation of TNBS (2.5 mg/animal) in 45% ethanol solution. The control mice received the 45% of ethanol solution. CTX (0.035 mg/kg) was administered i.p. 18 h after TNBS-induced colitis, and saline solution was administered as control. # *(p*<0.05) ETOH versus TNBS and TNBS+CTX; o *(p*<0.05) ETOH+CTX versus TNBS and TNBS+CTX; α *(p*<0.05) TNBS versus TNBS+CTX (n = 4–6 mice/group). **(B)** Disease activity index calculated as described in material and methods. The results were expressed as mean ± SEM (n = 4–6 mice/group); **(C)** Macroscopic appearance of colonic portion (4 cm) obtained from each mice/group at 4 days after TNBS-induced colitis; **(D)** Histological analysis of perirectal segment from mice of distinct experimental groups stained with H&E (Structures: (e) epithelial damage, (i) inflammatory infiltrate and (s) submucosa edema) obtained after 4 days of TNBS-colitis; **(E)** Histological score of inflammatory reaction perirectal segment of each experimental group of mice (n = 4–5 mice/group); **(F)** MPO activity of colonic tissue of each experimental mice-group. Groups: ETOH (control- 45% ETOH); ETOH+CTX (45% ethanol group treated with CTX); TNBS (TNBS instillation in 45% ETOH- inflammatory bowel disease) and TNBS+CTX (TNBS-instillation in 45% ETOH that received the CTX) (n = 4–5 animals/group). * *p*<0.05; *** *p*<0.001.

To address the modulatory effect of CTX on the intestinal inflammatory process, groups of mice received the intrarectal instillation of TNBS and after 18 h, the toxin was injected i.p. After 4 days of the TNBS-instillation, there was observed a higher percentage of weight loss and clinical score in the TNBS group compared with the control groups (ETOH or ETOH/CTX) (Fig 1A and 1B). A large necrotic area was also verified in the colon of the TNBS group (Fig 1C) confirming the establishment of the experimental colitis in the mice. In contrast, decreased weight loss, clinical score and necrotic area were observed in TNBS mice that received the CTX when compared with the untreated TNBS group (Fig 1A, 1B and 1C).

The histological analysis of the perirectal portion of TNBS mice showed loss of epithelial and goblet cells, crypt lesions and prominent transmural inflammatory cells infiltration in the intestinal mucosa and submucosa compared with mice receiving vehicle alone (ETOH) (Fig 1D and 1E). Moreover, we found that CTX administration in TNBS-mice resulted in lower histological signs of inflammation described above (Fig 1D and 1E). Thus, the TNBS-group treated with CTX showed similar histological scores as control groups (ETOH and ETOH/ CTX).

As a biomarker of local neutrophil infiltration, Fig 1F showed increased level of MPO in the TNBS group compared with the control groups. Furthermore, the CTX administration in TNBS-mice induced a decrease in this enzyme activity. Taken together, these results suggest that CTX was able to modulate the development of colitis induced by TNBS.

### Production of proinflammatory cytokines was down-modulated by CTX

**Fig 2:**
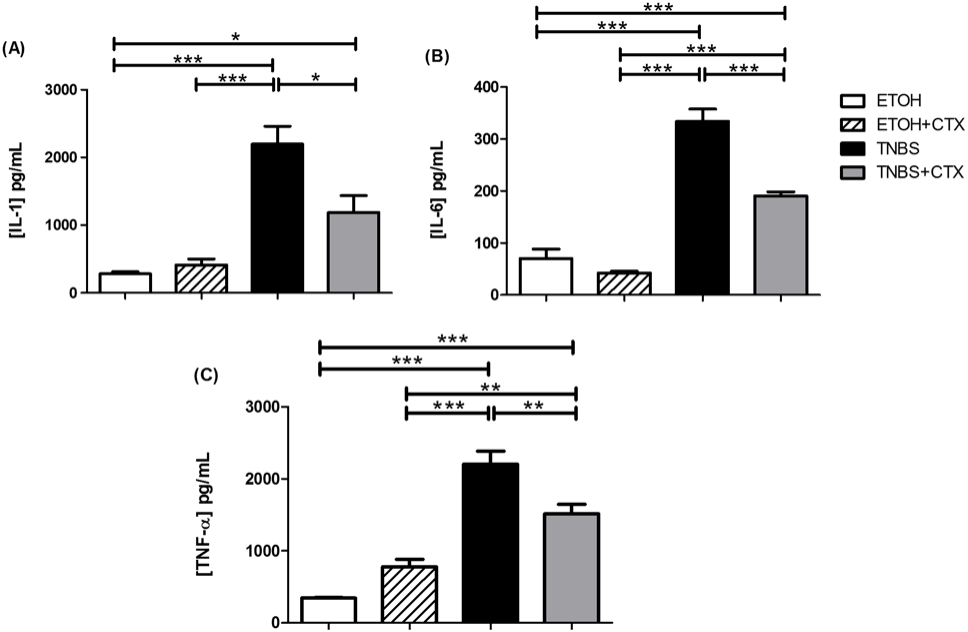
Secretion of pro-inflammatory cytokines in colonic tissue homogenates of TNBS-mice treated or not with CTX. Production of IL-1β (A), IL-6 (B) and TNF-α (C) was measured in homogenates of colonic segments by ELISA. The results represent the mean of the cytokine secretion in individual mice/group ± SEM. * *p*<0.05, ** *p*<0.01 and *** *p*<0.001 (n = 4–5 animals/group).

Considering that TNBS instillation in mice induced an intense colon inflammation, we analyzed the local secretion of IL-1β, IL-6 and TNF-α. The results showed high levels of these pro-inflammatory cytokines in homogenates of colonic tissue from the TNBS group compared with the control groups (ETOH and ETOH/CTX), reflecting the state of acute intestinal inflammation (Fig 2A, 2B and 2C). However, reduced secretion of these cytokines was observed in TNBS-mice that received CTX as compared with the untreated TNBS group (Fig 2A–2C).

### CTX down-modulated ILC3, Th17 cell population and IL-17 secretion in mice with TNBS-induced colitis

**Fig 3:**
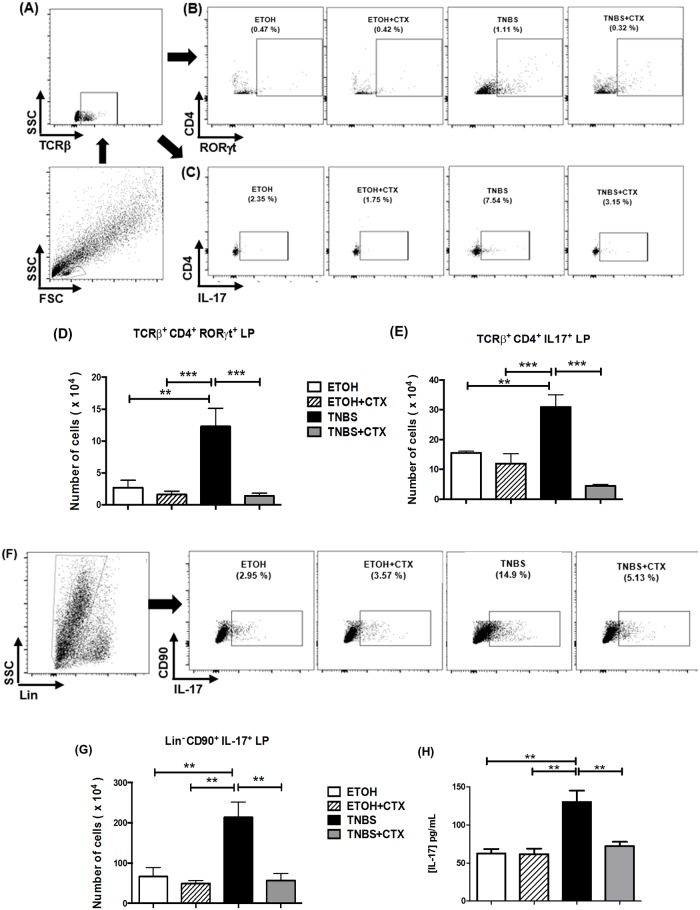
Effect of CTX on Th17 cells, ILC3 and IL-17 secretion of mice with acute colitis induced by TNBS. Cell suspensions were prepared from lamina propria of distinct experimental groups after 4 days of TNBS-induced colitis and that received or not the CTX. The TCRß^+^CD4^+^RORγt^+^, TCRß^+^CD4^+^IL-17^+^ and Lin^-^CD90^+^IL-17^+^ cells were analyzed by flow cytometry. **(A, B and C)** Strategy for the analysis of TCRß^+^CD4^+^RORγt^+^ and TCRß^+^CD4^+^IL-17^+^ cells in the lamina propria obtained from each group of mice. The results of TCRß^+^CD4^+^RORγt^+^ **(D)** and TCRß^+^CD4^+^IL-17^+^ cells **(E)** expressed as the mean of the absolute number ± SEM. The samples were prepared from a pool of cells from 4–5 animals/group performed in duplicate. The results are from 2–3 independent experiments. **(F)** Strategy for the analysis of the Lin^-^CD90^+^IL-17^+^ cells. **(G)** The results of Lin^-^CD90^+^IL-17^+^ cells were expressed as a mean of the absolute number ± SEM. The samples were prepared from a pool of cells from 4–5 animals/group performed in duplicate. The results are from 2 independent experiments. **(H)** Secretion of IL-17 in colonic tissue homogenates determined by ELISA. The results represent the mean obtained in the individual samples/group ± SEM. * *p*<0.05, ** *p*<0.01 and *** *p*<0.001 (n = 4–5 animals/group).

**Fig 4:**
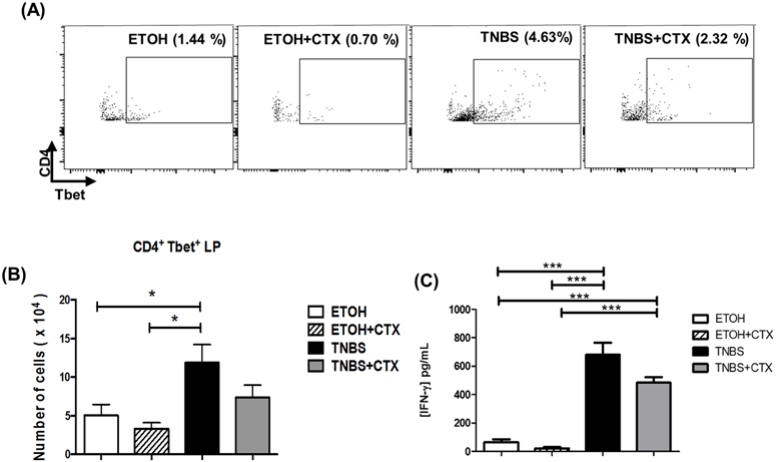
CD4^+^Tbet^+^ cell population and IFN-γ secretion of mice with acute colitis induced by TNBS treated or not with CTX. **(A)** Representative dot plots of CD4^+^Tbet^+^ cells in the lamina propria of distinct group of mice. **(B)** CD4^+^Tbet^+^ cells were expressed as a mean of the absolute number of cells ± SEM. The samples were prepared from a pool of cells from 4–5 animals/group performed in duplicate. The results are from 3 independent experiments. **(C)** Secretion of IFN-γ in colonic tissue homogenates determined by ELISA. The results represent the mean obtained in the individual samples/group ± SEM. * *p*<0.05, ** *p*<0.01 and *** *p*<0.001; (n = 4–5 animals/group).

As previously described T cell populations and ILCs are involved in the colonic inflammation. Consistent with this, we observed an increase in TCRß^+^CD4^+^RORγt^+^, TCRß^+^CD4^+^IL-17^+^ and Lin^-^CD90^+^IL-17^+^ cells in the lamina propria from the TNBS group when compared with the control groups (ETOH and ETOH/CTX) (Fig 3D, 3E and 3G). The CD4^+^Tbet^+^ cell population was also increased in the TNBS group compared with control groups (Fig 4B). In addition to this, high levels of IL-17 and IFN-γ were verified in colonic tissue homogenates of the TNBS group of mice (Fig 3H and 4C).

The Lin^-^CD90^+^IL-17^+^, TCRß^+^CD4^+^RORγt^+^ and TCRß^+^CD4^+^IL-17^+^ cell populations as well as IL-17 secretion were significantly lower in the TNBS-group that received the CTX in comparison with the untreated TNBS group (Fig 3D, 3E and 3G). Although, it was not statistically significant, the CD4^+^Tbet^+^ cell population and IFN-γ secretion were also lower when the TNBS-group received CTX (Fig 4B and 4C).

### CTX caused an increase in CD4^+^FoxP3^+^ cells and secretion of anti-inflammatory molecules in mice with TNBS-induced colitis

To determine whether the down-modulatory effect of CTX on acute inflammation could be correlated with the induction of an anti-inflammatory environment in mice, we analyzed the CD4^+^FoxP3^+^ cells in the lamina propria as well as IL-10 and TGF-β secretions in homogenates of colonic tissue from each mouse group. The PGE_2_ and LXA_4_ secretion was also analyzed, considering that these eicosanoids are involved in the modulatory effect of CTX in immune cells [42].

**Fig 5:**
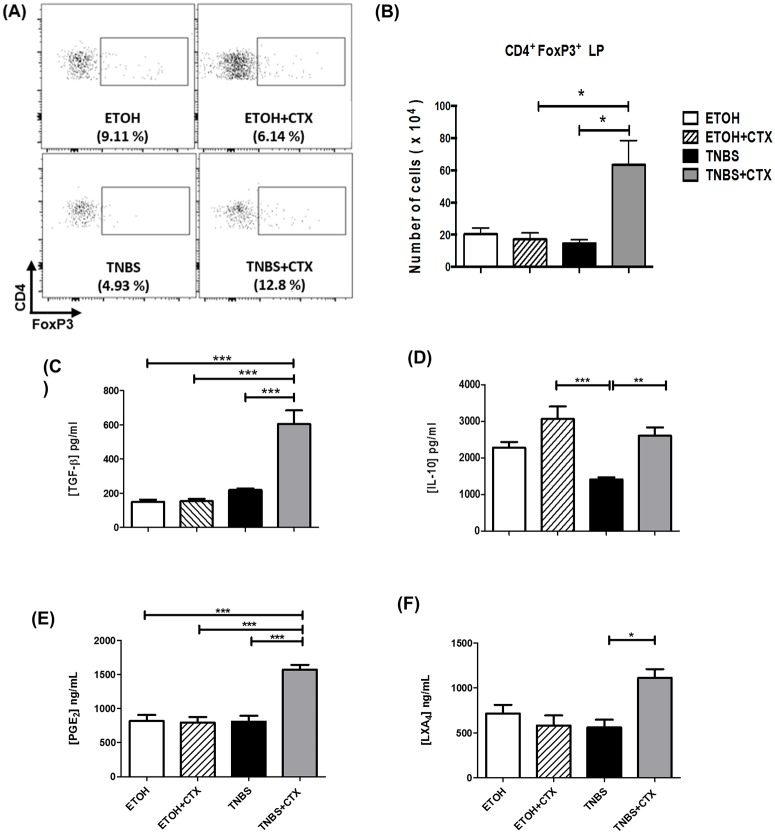
Analysis of CD4^+^FoxP3^+^ cells and anti-inflammatory mediators in TNBS-mice treated or not with CTX. Cell suspensions were prepared from lamina propria of distinct experimental groups after 4 days of TNBS-induced colitis for the analysis of CD4^+^FoxP3^+^ cells by flow cytometry. The samples were prepared from a pool of cells from 4–5 animals/group performed in duplicate. The results are from 2 independent experiments. **(A)** Representative dot plots of CD4^+^FoxP3^+^ cells in the lamina propria obtained from distinct experimental groups. **(B)** Results of CD4^+^FoxP3^+^ cells expressed as a mean of the absolute number of cells in duplicate of 2 independent experiments ± SEM. Secretion of TGF-β **(C)** and IL-10 **(D)** in colonic tissue homogenates determined by ELISA. Production of PGE_2_ **(E)** and LXA_4_ **(F)** was performed by ELISA in colonic tissue homogenates. The results represent the mean obtained in individual mice/group ± SEM. * *p*<0.05, ** *p*<0.01 and *** *p*<0.001; (n = 4–5 animals/group).

As shown in (Fig 5A, 5B and 5C) an increased CD4^+^FoxP3^+^ cell population in lamina propria as well as high production of TGF-β in colonic homogenates was detected in the TNBS group treated with CTX when compared with all other groups. Furthermore, we observed a lower production of IL-10 in the TNBS group compared with all other groups (Fig 5D).

The CTX-treated TNBS group also showed higher levels of PGE_2_ and LXA_4_ compared with the untreated TNBS group and controls (Fig 5E and 5F). Therefore, these results suggest that these anti-inflammatory mediators are involved in the effect of CTX on experimental inflammatory bowel disease.

## Discussion

The inappropriate and constant activation of the mucosal immune system is a critical event for the tissue injury observed in IBD. In this context, elements of innate immunity mediate intense inflammation and are involved in dysregulated cellular activation at different stages of the disease [58, 59, 60, 61]. Therefore, the elucidation of the immunological mechanisms involved in this process and the investigation of new therapies has been the subject of several studies [36, 55, 62].

Our data demonstrate the strong modulatory effect of CTX obtained from *C*.*d*.*terrificus* rattlesnake venom in the mouse experimental model of colitis induced by TNBS. Thus, the CTX was able to ameliorate the murine colitis elicited by intrarectal instillation of TNBS shifting the pro-inflammatory environment to local anti-inflammatory status.

Concerning other studies [63, 64], our data showed that the TNBS induced an acute colitis in BALB/c mice characterized by intense weight loss, clinical score, macroscopic signs, MPO activity, tissue injury and invasion of inflammatory cells infiltration. However, CTX administered after TNBS instillation in mice attenuated these signs of acute colitis.

Similar to that observed in our model, a protective effect of the crotapotin (CA), a CTX subunit, was reported in experimental models of autoimmune encephalomyelitis and neuritis resulting in a lower clinical score for these diseases [65, 66]. Other studies demonstrated that the CB, from CTX, exerts an anti-inflammatory effect in some experimental models [67, 68]. In addition, it was shown in several studies that the CTX (complex of CA and CB) has a potent modulatory role in distinct experimental models of immune system activation [46, 48, 49, 68, 69, 70, 71]. Our results provide evidences that the CTX can inhibit the innate immune response and consequent adaptive immunity.

As previously mentioned, in the activation of the intestinal immune system, increased secretion of pro-inflammatory cytokines and the recruitment of leukocytes are markers of the inflammatory response observed in IBD [72, 73, 74, 75, 76]. In agreement with these, IL-1β in combination with oxygen reactive species contributes to intestinal inflammation and the tissue damage [72]. Furthermore, the administration of anti-TNF-α antibody (infliximab) is effective in improving the clinical status of the patients with Crohn’s disease and the injection of anti-IL-6 receptor antibody in mice with colitis resulted in inhibition of IFN-γ, IL1-β mRNA as well as TNF-α secretion [77, 78].

Our data also showed high levels of IL-1β, IL-6 and TNF-α in homogenates of colonic segment from mice with TNBS-induced colitis. However, CTX administration resulted in the reduction of these pro-inflammatory cytokines secretion in TNBS-mice. Thus, the attenuated colon inflammation observed in the TNBS group that received CTX was most likely the result of the toxin’s ability to down-modulate the IL-1β, IL-6 and TNF-α secretion as well as the influx of inflammatory cells. In accordance with our data, it was earlier shown that the retinoic acid, which has an immunomodulatory effect, ameliorates the TBNS colitis via inhibition of TNF-α, IL-6, IL-1β secretion and MPO activity in mice [79].

ILCs have also been involved in the development of IBD by the secretion of IL-17A, IL-22 and IFN-γ in response to IL-23 [11, 80]. Along this line, the contribution of Lin^-^Thy^+^ILC3 secreting IL-17A was described in colitis of Tbet^-/-^Rag^-/-^ (TRUC) mice [81]. A predominance of ILC3 secreting IL-17A accompanied by few IFN-γ-expressing cells was also described in the Tbx21^-/-^Rag2^-/-^ ulcerative colitis (TRUC mice) disease model [81, 82]. In accordance with these findings, we also observed that TNBS induced an increase in ILC3 secreting IL-17A, which was prevented by the CTX administration.

In addition to innate immunity, distinct subsets of effector T cells such as Th1, Th2 and Th17 are increased and act in one or another variant of IBD [83, 84, 85, 86]. As a murine experimental model of human Crohn’s disease, Th1 and Th17 cells are involved in TNBS colitis [54, 55]. Our data also showed a prominent Th17 population and presence of Th1 cells, as well as high secretion of IL-17 and IFN-γ in TNBS-mice. However, CTX treatment in TNBS mice resulted in significant inhibition of Th17 cells and IL17 secretion but not an effect on Th1 response.

The crucial role of IL-17 in the development and severity of Crohn’s disease has been reported [87, 88]. It was shown that the IL-17 secretion by Th17 cells during the earlier phases (24–48 h) is critical to the development of the colitis, despite of the high IL-12 and IFN-γ production [54]. Furthermore, the blockage of the IL-17R-signaling attenuates the neutrophils migration and intestinal inflammation [54]. This effect of IL-17 was confirmed in another study describing lower neutrophil infiltration and inflammatory score in intestinal tissue in IL17^-/-^ mice with TNBS-induced colitis compared with IFN-γ^-/-^ or wild-type mice [89]. Therefore, these observations support the hypothesis that the inhibition of Th17 response induced by the CTX treatment in TNBS-mice could also be responsible for the lower local inflammatory cells migration and tissue damage observed in our experimental model.

The CTX treatment in TNBS-mice also induced a secretion of TGF-β, IL-10, PGE_2_ and LXA_4_ accompanied by CD4^+^FoxP3^+^ cells, suggesting their participation in the improvement of the colitis induced by TNBS. Supporting these findings, the immunomodulatory effect of a polysaccharide A from *Bacteroides fragilis* was directly correlated with TLR2-signalling and increased FoxP3^+^Treg cells secreting IL-10 in an experimental model of TNBS-induced colitis [90]. The role of FoxP3^+^ Treg cells preventing colitis mediated by Th1 and Th17 cells was also proven in an experimental model with the transfer of naive CD4 ^+^T cells to SCID mice [91]. In addition, the administration of anti-TGF-β antibodies in the SCID mice with co-transferred with FoxP3^+^ Treg and naive CD4 ^+^ T cells abolished the protective effect of the Treg cells, resulting in high levels of IL-17 and IFN-γ in the colon [91]. It was also reported in an experimental TNBS-colitis model in SJL/J mice that the transfer of Treg cells prevents the establishment of the disease and that this protective effect is abolished by the administration of anti-IL-10 and/or anti-TGF-β antibodies [92].

In another study, it was reported that the attenuated colitis in mice treated with hyaluronic acid was directly correlated with the increased expression of cyclooxygenase 2 (Cox2) and PGE_2_ production via a TLR4-dependent mechanism [93]. PGE_2_ was also shown to be related with the induction of FoxP3 expression by CD4^+^CD25^-^ and CD4^+^CD25^+^ cells as well as the inhibition of IL-17 secretion and RORγt expression by CD4^+^CD62L^+^ cells cultured with TGF-β and IL-6 [94, 95]. Moreover, the administration of a LXA_4_ analog was found to induce IL-10 secretion and to prevent the tissue damage, TNF-α secretion and lethality of mice subjected to intestinal ischemia/reperfusion [96].

The induction of PGE_2_ and LXA_4_ by CTX or CB subunit was previously described in cultures of resident or elicited macrophages from peritoneal cavity of rats. It was also shown that LXA_4_ was involved in the inhibitory effect of CTX on the phagocytic activity of macrophages *in vitro* [49]. Furthermore, LXA_4_ induced by CTX can be correlated with the inhibition of neutrophil migration to the peritoneal cavity induced by carrageenan injection or paw edema in mice injected with Bacillus Calmette-Guérin (BCG) [50, 97]. In accordance with these reports, our data suggest that CTX induces an anti-inflammatory status in mice able to control the intestinal inflammation induced by the TNBS.

## Conclusion

Our study demonstrated that CTX has a potent immunomodulatory effect *in vivo*, considering that it was able to attenuate the colitis induced by a chemical agent as the TNBS in mice. The mechanism involved in this CTX effect includes the down-modulation of leukocytes migration, ILC3 and Th17 responses and secretion of IL-1β, TNF-α, IL- 6 and IL- 17. This effect is accompanied by increased secretion of TGF-β, PGE_2_ and LXA_4_ and the presence of CD4^+^FoxP3^+^ cells.

## Supporting Information

S1 File Data from body weight, clinical score, histological analysis, MPO activity, cytokine secretion and cell populations of BALB/c mice with TNBS-induced colitis treated or not with CTX. (PDF)
