# Supplementary material for: Crotoxin from Crotalus durissus terrificus Is Able to Down-Modulate the Acute Intestinal Inflammation in Mice
Source: PLoS One. 2015 Apr 8;10(4):e0121427. doi: 10.1371/journal.pone.0121427 (PMC4390225; doi:10.1371/journal.pone.0121427)
Supplement: S1 File — (PDF) [file pone.0121427.s001.pdf]

Table 1- Body weight (g) of BALB/c mice during four days after the TNBS-induced colitis treated or not with CTX

Groups	ETOH			
Mice	Day 1	Day 2	Day 3	Day 4
1	25.30	25.30	25.10	25.00
2	25.80	25.60	25.70	25.80
3	25.70	25.30	26.00	26.10
4	25.50	25.60	25.50	25.63
**Mean**	25.58	25.45	25.58	25.63
**SEM**	0.11	0.09	0.19	0.23

	ETOH+CTX			
Mice	Day 1	Day 2	Day 3	Day 4
1	24.50	24.40	24.00	23.40
2	25.60	25.40	24.50	24.40
3	25.60	25.30	25.10	25.60
4	25.00	25.20	24.00	25.00
Mean	25.18	25.08	24.40	24.60
SEM	0.27	0.23	0.26	0.47

	TNBS			
Mice	Day 1	Day 2	Day 3	Day 4
1	26.40	22.70	21.20	19.70
2	27.90	23.60	21.30	20.20
3	25.10	21.50	20.20	18.80
4	26.90	22.80	20.10	18.70
5	26.20	22.60	21.50	20.80
6	25.40	22.30	21.90	20.10
**Mean**	26.32	22.58	21.03	19.72
**SEM**	0.42	0.28	0.30	0.34

	TNBS+CTX			
Mice	Day 1	Day 2	Day 3	Day 4
1	26.10	22.60	22.90	23.10
2	26.40	24.70	24.50	24.30
3	25.90	23.20	23.70	23.40
4	27.20	23.00	24.70	24.70
5	25.70	23.40	22.90	23.00
6	26.70	23.50	23.30	22.30
Mean	26.33	23.40	23.67	23.47
SEM	0.23	0.29	0.32	0.36

Table 2- Percentage of weight of BALB/c mice during four days after the TNBS-induced colitis treated or not with CTX

Group	ETOH			
Mice	Day 1	Day 2	Day 3	Day 4
1	100	100	99.2	98.8
2	100	99.2	99.6	100
3	100	98.4	100.2	100.6
4	100	100.4	100	100.5
**Mean**	100	99.5	100	100.2
**SEM**	0	0.4	0.42	0.6

Group	ETOH+CTX			
Mice	Day 1	Day 2	Day 3	Day 4
1	100	99.6	97.9	95.5
2	100	99.2	95.7	95.3
3	100	98.8	98.1	100
4	100	100	96	100
**Mean**	**100**	**99.6**	**96.9**	**97.7**
**SEM**	**0**	**0.43**	**0.63**	**1.3**

Group	TNBS			
Mice	Day 1	Day 2	Day 3	Day 4
1	100	86	80.3	74.6
2	100	84.6	76.3	72.4
3	100	85.7	80.5	74.9
4	100	84.8	74.7	69.5
5	100	86.3	82.1	79.4
6	100	87.8	86.2	79.1
**Mean**	100	85.8	80.0	75
**SEM**	0	0.5	1.7	1.6

Group	TNBS+CTX			
Mice	Day 1	Day 2	Day 3	Day 4
1	100	86.6	87.7	88.5
2	100	93.6	92.8	92.1
3	100	89.6	91.5	90.4
4	100	84.6	90.8	90.8
5	100	91.1	89.1	89.5
6	100	88.0	87.3	83.5
**Mean**	100	88.9	89.9	89.1
**SEM**	0	1.3	0.9	1.2

Table 3- Clinical score of BALB/c mice during four days after the TNBS-induced colitis treated or not with CTX

Group	ETOH			
Mice	Day 1	Day 2	Day 3	Day 4
1	0.00	0.00	0.00	1.00
2	0.00	0.00	0.00	0.00
3	0.00	3.00	1.00	0.00
4	0.00	0.00	0.00	0.00
**Mean**	0.00	0.75	0.25	0.25
**SEM**	0.00	0.75	0.25	0.25

Group	ETOH+CTX			
Mice	Day 1	Day 2	Day 3	Day 4
1	0.00	0.00	1.00	1.00
2	0.00	2.00	1.00	1.00
3	0.00	2.00	1.00	0.00
4	1.00	2.00	5.00	2.00
**Mean**	0.25	1.50	2.00	1.00
**SEM**	0.25	0.50	1.00	0.41

Group	TNBS			
Mice	Day 1	Day 2	Day 3	Day 4
1	0.00	6.00	8.00	8.00
2	0.00	8.00	8.00	8.00
3	0.00	4.00	8.00	8.00
4	0.00	8.00	8.00	8.00
5	0.00	8.00	8.00	6.00
6	0.00	6.00	8.00	8.00
**Mean**	0.00	6.67	8.00	7.67
**SEM**	0.00	0.67	0.00	0.33

Group	TNBS+CTX			
Mice	Day 1	Day 2	Day 3	Day 4
1	0.00	7.00	5.00	3.00
2	0.00	4.00	2.00	2.00
3	0.00	6.00	2.00	2.00
4	0.00	4.00	3.00	5.00
5	0.00	5.00	2.00	2.00
6	0.00	3.00	3.00	2.00
**Mean**	0.00	4.83	2.83	2.67
**SEM**	0.00	0.60	0.48	0.49

Table 4- Histological analysis of perirectal segment and MPO activity of BALB/c mice with TNBS-induced colitis treated or not with CTX

	**Histological score - Groups**			
**Mice**	**ETOH**	**ETOH+CTX**	**TNBS**	**TNBS+CTX**
1	2.30	1.70	8.00	0.70
2	1.00	0.00	7.00	0.00
3	1.00	1.70	8.00	0.70
4	1.30	1.20	7.00	4.70
5	-	-	7.00	1.40
**Mean**	1.40	1.15	7.40	1.50
**SEM**	0.31	0.40	0.24	0.83

	**MPO activity - Groups**			
**Mice**	**ETOH**	**ETOH+CTX**	**TNBS**	**TNBS+CTX**
1	0.005	0.003	0.036	0.007
2	0.005	0.002	0.044	0.010
3	0.006	0.005	0.045	0.010
4	0.008	0.006	0.030	0.005
5	-	-	0.048	0.015
**Mean**	0.006	0.004	0.041	0.009
**SEM**	0.001	0.001	0.003	0.002

Table 5- IL-1 β and TNF-α production in homogenates of colonic segments from mice with TNBS-induced colitis treated or not with CTX

IL-1β (ng/mL)				
Mice	ETOH	ETOH+CTX	TNBS	TNBS+CTX
1	377.1	500.4	2312.0	391.4
2	237.2	600.0	2186.4	1294.8
3	227.7	201.7	2738.9	1885.2
4	280.7	356.3	2539.7	1389.6
5	-	-	1198.5	988.1
Mean	280.7	414.6	2195.1	1189.8
SEM	34.6	86.8	266.6	246.2

TNF-α (ng/mL)				
Mice	ETOH	ETOH+CTX	TNBS	TNBS+CTX
1	358.5	866.8	2010.7	1702.0
2	353.0	503.7	2283.1	1393.4
3	328.0	975.8	1829.1	1211.8
4	346.0	776.1	2864.1	1919.9
5	-	-	2047.0	1357.1
Mean	346.4	780.6	2206.8	1516.8
SEM	6.6	100.9	179.5	128.6

IL-6 (ng/mL)				
Mice	ETOH	ETOH+CTX	TNBS	TNBS+CTX
1	66.8	36.4	345.2	190.8
2	57.6	51.6	381.4	209.0
3	33.4	39.4	321.0	202.0
4	121.2	42.0	372.4	187.8
5	-	-	251.4	163.6
Mean	69.8	42.4	334.3	190.6
SEM	18.5	3.3	23.3	7.8

Results of cytokine production obtained in individual samples/group.

Table 6- IL-17 and IFN-γ production in colonic tissue homogenates from mice with TNBS-induced colitis treated or not with CTX

IL-17 (ng/mL)				
Mice	ETOH	ETOH+CTX	TNBS	TNBS+CTX
1	75.9	74.2	121.5	84.9
2	52.8	42.1	103.5	81.5
3	52.8	57.8	100.7	69.7
4	69.0	72.0	145.8	71.9
5	-	-	180.0	54.5
Mean	62.6	61.5	130.3	72.5
SEM	5.9	7.4	14.8	5.3

IFN-γ (ng/mL)				
Mice	ETOH	ETOH+CTX	TNBS	TNBS+CTX
1	13.9	15.6	557.0	397.0
2	109.0	45.0	680.0	460.0
3	77.0	15.6	435.0	625.0
4	63.0	15.1	889.0	480.0
5	-	-	839.0	460.0
Mean	65.7	22.8	680.0	484.4
SEM	19.8	7.4	84.9	37.8

Results of cytokine production obtained in individual samples/group.

Table 7- Cell populations analyzed in lamina propria from mice with TNBS-induced colitis treated or not with CTX

	$TCR\beta^+CD4^+ROR\gamma T^+$	$TCR\beta^+CD4^+IL\text{-}17^+$	$LIN^-CD90^+IL\text{-}17^+$	$CD4^+Tbet^+$	$CD4^+FoxP3^+$
**ETOH**	2.68 ±1.07	15.51 ± 0.61	60.91 ± 22.12	5.02 ± 1.33	20.29 ± 3.75
**ETOH+CTX**	1.58 ± 0.47	11.97 ± 3.31	49.45 ± 7.37	3.25 ± 0.71	17.15 ± 3.50
**TNBS**	12.29 ± 2.59	30.90 ± 4.15	213.50 ± 37.99	12.0 ± 2.10	14.66 ± 1.98
**TNBS+CTX**	1.39 ± 0.38	4.42 ± 0.58	56.83 ± 12.50	7.34 ± 1.48	61.22 ± 14.49

The results ($x10^4$) represent the mean ± SEM obtained from cell suspensions prepared from 4-5 mice/group in duplicate and 2-3 experiments performed independently.

Table 8- TGF-β and IL-10 production in colonic tissue homogenates of mice with TNBS-induced colitis treated or not with CTX

TGF-β (pg/mL)					IL-10 (pg/mL)				
**Mice**	**ETOH**	**ETOH+CTX**	**TNBS**	**TNBS+CTX**	**Mice**	**ETOH**	**ETOH+CTX**	**TNBS**	**TNBS+CTX**
1	170.1	148.2	218.2	734.6	1	2141.0	3836.6	1615.8	2208.6
2	130.7	117.6	196.3	822.1	2	2651.2	2846.2	1435.8	2448.6
3	175.0	170.1	248.0	563.9	3	1931.0	3341.4	1398.4	2763.8
4	124.0	180.0	205.1	533.3	4	2394.0	2216.2	1330.8	2201.0
5	-	-	229.0	371.4	5	-	-	1263.4	3409.0
**Mean**	149.9	154.0	219.3	605.1	**Mean**	2279.3	3060.1	1408.8	2606.2
**SEM**	13.2	13.8	9.1	79.2	**SEM**	156	346.4	59.5	225.4

Results of cytokine production detected in individual samples/ experimental group.

Table 9- PGE_2 and LXA_4 production in colonic tissue homogenates of mice with TNBS-induced colitis treated or not with CTX

PGE_2					LXA_4				
**Mice**	**ETOH**	**ETOH+CTX**	**TNBS**	**TNBS+CTX**	**Mice**	**ETOH**	**ETOH+CTX**	**TNBS**	**TNBS+CTX**
1	862.9	739.7	646.3	1380.8	1	536.6	425.4	336.2	815.8
2	935.5	877.2	883.6	1567.5	2	561.4	409.3	580.4	994.1
3	722.8	761.7	874.5	1933.3	3	853.8	587.7	795.2	1386.0
4	870.7	693.0	923.8	1632.4	4	909.3	897.6	684.2	1125.7
5	-	-	835.6	1759.5	5	-	-	406.4	1233.9
**Mean**	847.97	767.9	832.8	1654.7	**Mean**	715.3	580.0	560.5	1111.1
**SEM**	44.79	39.1	48.7	92.7	**SEM**	96.8	113.3	85.1	97.9

Results of PGE_2 and LXA_4 production detected in individual samples/experimental group.
